# Preoperative Sarcopenia in Predicting Postoperative Complications following Gastrointestinal Cancer Surgery: An Observational Study

**DOI:** 10.31729/jnma.9107

**Published:** 2025-07-01

**Authors:** Narendra Maharjan, Paleswan Joshi Lakhey, Sumita Pradhan, Bishnu Prasad Kandel, Ramesh Singh Bhandar

**Affiliations:** 1 Department of Surgical Gastroenterology, Tribhuvan University Teaching Hospital, Maharajgunj, Kathmandu, Nepal

**Keywords:** *nutrition risk index*, *postoperative complication*, *sarcopenia*

## Abstract

**Introduction:**

Surgery is the mainstay for the management of gastrointestinal cancer patients, but it is associated with various complications. Sq identification cf pre-operative factors associated with post-operative complications is essential. Sarcopenia is increasingly recognized as a prognostic factor of post-operative complications following various cancer surgeries. This study aimed to determine whether precperative sarcopenia is associated with postoperative complications following gastrointestinal cancer surgery.

**Methods:**

A prospective observational study was conducted from April 2019 to August 2020 at Tribhuvan University Teaching Hospital, Nepal. Eighty-nine patients undergoing elective curative gastrointestinal cancer surgery were included in the study. Patients less than 16 years and who did not undergo curative resections were excluded. Sarcopenia was defined using the Skeletal Muscle Index (SMI), which was measured via abdominal computed tomography imaging. Postoperative complications were graded using the Clavien-Dindo classification. Statistical analyses were performed to evaluate the associations of preoperative sarcopenia with postoperative complications using SPSS version 23.

**Results:**

Among 89 patients, the mean age was 55.6±13 years, 48 (53.93%) were male, and sarcopenia was present in 36 (40.45%) patients. Clavien-Dindo ≥3 level complication occurred in 17 (47.22%) patients with sarcopenia.

**Conclusions:**

Almost half of the patient with sarcopenia developed post operative complications.

## INTRODUCTION

Gastrointestinal (GI) cancer surgery is increasing in this era because diagnostic techniques and management protocols have evolved.^1^ Surgery is the backbone in the management of GI cancer patients. However, surgery is associated with various complications, which can lead to decreased tolerance to adjuvant therapy and poor survival outcomes.^2, 3^

It is essential to decrease post-operative complications, for which a good predicting factor is crucial. Patient's frailty is a strong predictor of post-operative complications, but not accurately defined by traditional determinants.^4^ Sarcopenia, a hallmark of frailty, is a good predictor of post-operative complications.^5-7^ The prevalence of sarcopenia increases with advanced age.^8^ Sarcopenia refers to the loss of skeletal muscle mass, muscle strength, and physical performance.^9^ It is found to be an independent factor determining poor outcomes after various onco-surgeries, but not studied in Nepalese population.^10,11^ This study aimed to find the proportion of post-operative complication in patient with sarcopenia undergoing gastrointestinal cancer surgery.

## METHODS

This prospective observational study was conducted at the Department of GI and General Surgery, Tribhuvan University Teaching Hospital (TUTH), Kathmandu, Nepal, from April 2019 to August 2020. Tribhuvan University Teaching Hospital is a tertiary care referral centre and a University Teaching Hospital. The accessible population was patients undergoing gastrointestinal cancer surgery at TUTH. The sample population was all eligible patients undergoing surgery between April 2019 to August 2020. Patients more than or equal to 16 years providing consent were included in the study, while those who had not received curative resections were excluded from the study. Ethical approval was received from the Institutional Review Committee on April 26, 2019 [Reference number: 438 (6-11)]. Data collection was started after hospital admission of the patients using a proforma and recorded in Microsoft Excel (Microsoft Office 2019). Informed consent was taken from all patients included in the study. Demographic and clinical data like age, sex, Body Mass index (BMI), serum albumin, Eastern Cooperative Oncology Group Performance Status (ECOG), American Society of Anesthesiologists physical status (ASA), Nutrition Risk Index (NRI), presence of sarcopenia, and postoperative complications were collected.

The presence of sarcopenia was defined using the skeletal muscle index (SMI), calculated from preoperative Computed Tomography (CT) scans. The CT scans performed within 30 days before the operation were used for the analysis; two adjacent axial CT images were selected at the level of the third lumbar vertebra (L3). The CT scans could be either contrast-enhanced or unenhanced and must have a slice thickness of at least 5 mm. Skeletal muscle area was quantified at a Hounsfield Unit (HU) range of -29 to +150. The Skeletal Muscle Index (SMI) was calculated using the formula: SMI = (skeletal muscle area at L3) / (height in meters)^2^. Sarcopenia was defined as an SMI of less than 43 cm^2^/m^2^ for men with a body mass index (BMI) < 25 kg/m^2^, and less than 53 cm^2^/m^2^ for men with a BMI ≥ 25 kg/m^2^. For women, sarcopenia was defined as an SMI of less than 41 cm^2^/m^2^, regardless of BMI.^12^

All patients were kept nil per oral from midnight before surgery, and maintenance intravenous (IV) fluids were started the night prior. Prophylactic antibiotics (e.g., ceftriaxone 1 gm IV) were given 30 minutes before skin incision. Postoperatively, patients received IV fluids, analgesics, and antibiotics as per the surgeon's discretion. Abdominal drains and nasogastric tubes were used as required. Patients were managed in the postoperative ward or Intensive Care Unit (ICU) until stable, then transferred to the ward as per the decision of the operating surgeon.

The post-operative period in this study refers to 30 days post-surgery, and complications that occurred during this period were recorded to be classified according to Clavien-Dindo classification,^13^ in which major complications were defined as grade ≥ 3A, and overall complications as grade I to V. The ASA and ECOG performance scores were used to evaluate the patient's physical fitness for surgery. The. NRI was calculated using formula: NRI = NRI = NRI = (1.519 × serum albumin, g/dL) + {41.7 × present weight (kg)/ ideal body weight(kg)}.^14^

Statistical analyses were conducted using IBM SPSS Statistics for Windows, version 23 (IBM Corp., Armonk, N.Y., USA). Continuous variables were reported as proportions, meant standard deviation (SD) or median with ranges, depending on the distribution.

## RESULTS

There were 121 patients evaluated during the study duration. Of these, 32 (26.45%) patients were excluded from the study: 28 (23.14%) due to non-curative resections and 4 (3.30%) due to benign pathologies. Thus, 89 patients were included in the study. The proportion of sarcopenia in this study was 36 (40.45%). Periampullary carcinoma was 36 (40.45%), colorectal carcinoma 22 (24.72%), gastric carcinoma 18 (20.22%), and hepatobiliary carcinoma 13 (14.61%). The mean age of the patients was 55.6 ± 13.0 years (range: 18-79 years). There were 48 (53.9%) males and 41 (46.1%) females. The mean Body Mass Index (BMI) was 21.98 ± 3.27 kg/m^2^ (Table 1). Clavien-Dindo grade ≥3A complications were reported in 17 (47.22%) patients with sarcopenia and 11 (20.75%) patients without sarcopenia (Table 2). Among patients with overall post-operative complications, the mean BMI was 21.05±3.42 in the sarcopenia group and 22.86±2.95 in the no sarcopenia group. For Clavien-Dindo grade ≥3A complications, ECOG performance status 2 was recorded in 4 (23.53%) patients with sarcopenia and 1 (9.09%) without sarcopenia. (Table 3).

**Table 1:** Demographic and clinical characteristics of the patients undergoing gastrointestinal cancer surgery (n=121).

Variables	All patients (n=89)	Patients without sarcopenia (n=53)	Patient with sarcopenia (n=36)
Age, years (mean±SD)	55.60±12.96	54.26±12.36	57.56±13.37
Sex			
Female n(%)	41(46.07)	25(47.17)	16(44.44)
Male n(%)	48(53.93)	28(52.83)	
BMI (mean±SD)	21.98±3.27	22.50±3.11	21.21±3.35
ECOG n(%)			
0	34 (38.20)	22(41.51)	12(33.33)
1	49(55.06)	30(56.60)	19(52.78)
2	6(6.74)	1(1.89)	5(13.89)
ASA, n(%)			
1	78(87.64)	48(90.57)	30(83.33)
2	9(10.11)	4(7.55)	5(13.89)
3	2(2.25)	1(1.89)	1(2.78)
Albumin (mean±SD)	37.20±6.63	38.39±5.54	35.44±7.64
NRI (mean ±SD)	93.11±13.81	94.55±13.37	90.93±14.18

SD=Standard Deviation; BMI=Body Mass Index; ECOG= Eastern Cooperative Oncology Group; NRI= Nutrition Risk Index; ASA= American Society of Anesthesiologists physical status; NRI= Nutrition Risk Index.

**Table 2:** Postoperative Complications in patients with and without sarcopenia undergoing gastrointestinal cancer surgery (n=89).

Variables	Sarcopenia (n=36)	No Sarcopenia (n=53)
Post-operative complications (n=63)	28(77.78%)	35(66.04%)
Clavien Dindo ≥ 3A n(%)	17(47.22)	11(20.75)
Clavien Dindo < 3A n(%)	11(30.55)	24(45.28)
No post-operative complications (n=26)	8(22.22%)	18(33.96%)

**Table 3:** Comparison of clinical variables in patients with and without sarcopenia undergoing gastrointestinal cancer surgery (n=89).

Variables	Sarcopenia		No Sarcopenia	
	Overall Post-Operative Complications (n=28)	Clavien Dindo ≥ 3A (n=17)	Overall Post- Operative Complications (n=35)	Clavien Dindo > 3A (n=11)
Age, years (mean ±SD)	57.36±14.18	55.88±14.97	55.77±11.43	59.7±12.15
Gender				
Male n(%)	14(50)	8(47.06%)	20(57.14)	7(63.64)
Female n(%)	14(50)	9(52.94)	15(42.86)	4(36.36)
BMI (mean±SD)	21.05±3.42	21.03±3.19	22.86±2.95	21.68±1.57
ECOG, n(%)				
0	8(28.57)	5(29.41)	17(48.57)	4(36.36)
1	15(53.57)	8(47.06)	17(48.57)	6(54.55)
2	5(17.86)	4(23.53)	1(2.86)	1(9.09)

## DISCUSSION

Sarcopenia was present in 36 (40.45%) patients in this cohort. Among them, 28 (77.78%) patients had overall post-operative complications, and 8 (22.22%) patients did not have complications. The mean age of the sarcopenic patients who had the complications was 57.36 year ±14.18, which did not differ from non- sarcopenic patients having the complications. Also, gender and BMI were similar in both groups. There were 17 (47.22%) sarcopenic patients who had major post-operative complications, and their mean age was 55.88 years ± 14.97. Among them, 8 (47.06%) patients were male. Buettner et al found that BMI and gender were similar in patients with or without sarcopenia, but found sarcopenia to be more prevalent in older patients.^1^

There are various methods of assessing the nutritional and performance status of the patients pre-operatively, like Body Mass Index, Serum albumin, Nutrition Risk Index, ECOG, ASA, and sarcopenia. The World Health Organization has defined the ideal BMI range for the Asian population (23 - 27.5 kg/m2) slightly lower than that for the European population (25 - 29.9 kg/m2).^15^ This is because the Asian people have a relatively higher risk of type 2 diabetes and cardiovascular disease at BMIs lower than the existing WHO cut-off point for overweight (> or =25 kg/m2). Patients having the same BMIs can have different SMIs and vice versa.^12^ This shows that there is a poor correlation between BMI and SMI. This might be the reason that we did not find differences in the BMI of patients with and without sarcopenia.

Serum albumin is usually considered to be a predictor of postoperative outcome following various GI cancer surgeries.^16-18^ Though we found preoperative serum albumin was lower in patients with post-operative complications, the difference was insignificant. Also, the serum albumin level was similar in both patients with and without sarcopenia. This is similar to that observed by Mercan U et al in their study.^19^

The Nutrition Risk Index (NRI) was developed by the Veterans Affairs Total Parenteral Nutrition Cooperative Study Group.^20^ NRI was similar in patients with and without sarcopenia in this study. However, a study had shown that low NRI was associated with sarcopenia.^21^

Eastern Cooperative Oncology Group (ECOG) performance status included grades from 0 to 4, where 0 defines a fully active and 4 a completely disabled patient.^22^ ECOG performance status subjectively measures the functional status of a patient. Also, patients having higher ECOG performance scores were found to have sarcopenia.^19^ However, we found ECOG performance status was similar in patients with or without sarcopenia. Most of the patients (93.3%) had an ECOG performance score of 0 to 1, and only 6.7% of patients had an ECOG performance score of 2. None of them had a score of more than 2. This showed that we selected patients having good performance scores for the operation.

The American Society of Anesthesiologists (ASA) physical status is a commonly used method of identifying a patient's fitness for surgery. It had undergone many modifications to improve its predictive value for patients' physical fitness. It has been criticised due to its subjectivity and interobserver variability.^23^ Sarcopenic patients were found to have a higher ASA score.^19^ However, we had similar ASA scores in patients with or without sarcopenia. This may be due to the subjective nature of the ASA scoring system and selection bias, as patients having good performance status were usually selected for the surgery.

Mac Donald Critchley (1931) was the first to describe that muscle mass decreases with aging.^24^ Later, Rosenberg coined the term "sarcopenia".^25^ Then, many studies were conducted regarding the utility of sarcopenia in determining a patient's physical fitness and postoperative complications. However, there were challenges in determining the accurate cut-off value and the method used to identify the presence of sarcopenia. So, there were different methods of determining muscle mass, like Dual-energy X-ray absorptiometry (DXA), Bioimpedance analysis, CT scan, and MRI. Also, there were different methods of identifying muscle strength, such as Knee flexion/ extension, Handgrip strength, and Peak expiratory flow. Similarly, different methods of identifying physical performance status, like the timed get-up-and- go test, usual gait speed, Short Physical Performance Battery (SPPB), and Stair Climb Power Test.^9^ The various working groups (European, International, Asian) on sarcopenia provided defining criteria and diagnostic techniques.^9,26,27^ We have used a CT scan of the abdomen to calculate skeletal muscle area at the level of the 3rd lumbar vertebra, which is divided by the patient's height to get skeletal muscle index. This is because we routinely do a CT abdomen to stage the disease in gastrointestinal cancer patients. So, it will not add any economic burden to the patients. This SMI would decide the presence of sarcopenia according to the cut-off value given by Martin L et al.^12^ Buettner S et al. had demonstrated that sarcopenic patients had a higher prevalence of major postoperative complications.^1^ We also found that sarcopenic patients had a higher major postoperative complications rate, but not overall postoperative complications. There are several limitations of this study. First, surgeries were performed by different surgeons, introducing variability in techniques and postoperative care. Second, the study included patients with different types and stages of GI cancers, which may prevent the generalizability. Third, there may be an inter-observer variability in the measurement of skeletal muscle area from CT images.

## CONCLUSIONS

Half of the patient with sarcopenia developed post operative complications. This was consistent with other study.
